# A One Health view of the West Nile virus outbreak in Andalusia (Spain) in 2020

**DOI:** 10.1080/22221751.2022.2134055

**Published:** 2022-10-26

**Authors:** Jordi Figuerola, Miguel Ángel Jiménez-Clavero, María José Ruíz-López, Francisco Llorente, Santiago Ruiz, Andreas Hoefer, Pilar Aguilera-Sepúlveda, Jéssica Jiménez-Peñuela, Olaya García-Ruiz, Laura Herrero, Ramón C. Soriguer, Raúl Fernández Delgado, María Paz Sánchez-Seco, Josué Martínez-de la Puente, Ana Vázquez

**Affiliations:** a Estación Biológica de Doñana – CSIC, Sevilla, Spain; b Centro de Investigación en Sanidad Animal (CISA-INIA), CSIC, Valdeolmos, Spain; c Servicio de Control de Mosquitos de la Diputación Provincial de Huelva, Ctra. Hospital Infanta Elena, Huelva, Spain; d Centro Nacional de Microbiología, Instituto de Salud Carlos III, Majadahonda, Spain; e Departamento de Parasitología, Universidad de Granada, Granada, Spain; f CIBER de Enfermedades Infecciosas (CIBERINFEC), Madrid, Spain; g CIBER de Epidemiología y Salud Publica (CIBERESP), Madrid, Spain; h European Public Health Microbiology Training Programme (EUPHEM), European Centre for Disease Prevention and Control (ECDC), Stockholm, Sweden

**Keywords:** *Culex*, birds, epizootic transmission, flavivirus, mosquitoes, West Nile virus, vector-borne diseases, Zoonoses

## Abstract

Reports of West Nile virus (WNV) associated disease in humans were scarce in Spain until summer 2020, when 77 cases were reported, eight fatal. Most cases occurred next to the Guadalquivir River in the Sevillian villages of Puebla del Río and Coria del Río. Detection of WNV disease in humans was preceded by a large increase in the abundance of *Culex perexiguus* in the neighbourhood of the villages where most human cases occurred. The first WNV infected mosquitoes were captured approximately one month before the detection of the first human cases. Overall, 33 positive pools of *Cx. perexiguus* and one pool of *Culex pipiens* were found. Serology of wild birds confirmed WNV circulation inside the affected villages, that transmission to humans also occurred in urban settings and suggests that virus circulation was geographically more widespread than disease cases in humans or horses may indicate. A high prevalence of antibodies was detected in blackbirds (*Turdus merula*) suggesting that this species played an important role in the amplification of WNV in urban areas. *Culex perexiguus* was the main vector of WNV among birds in natural and agricultural areas, while its role in urban areas needs to be investigated in more detail. *Culex pipiens* may have played some role as bridge vector of WNV between birds and humans once the enzootic transmission cycle driven by *Cx. perexiguus* occurred inside the villages. Surveillance of virus in mosquitoes has the potential to detect WNV well in advance of the first human cases.

## Introduction

The number of newly emerging infectious diseases has increased considerably in recent years and poses one of the major global health challenges due to the devastating consequences for human populations and the economy [1]. Vector-borne diseases represent one in four of the newly emerging infectious diseases [2]. West Nile Virus (WNV; *Flaviviridae* family*, Flavivirus* genus) is an important vector-borne disease distributed worldwide [3], and is the principal causative agent of viral encephalitis in humans, with a considerable impact both on public and animal health [4]. The virus is maintained in nature in an enzootic cycle involving ornithophilic mosquitoes which are the transmission vectors and many species of birds that are reservoir hosts [5]. The virus can infect over 300 species of birds only in North America, but there is a wide range of variation between species in the susceptibility to WNV [6]. As a consequence, the role each bird species plays in viral transmission, amplification, and outbreaks vary greatly [6]. Several mosquito genera are also competent for WNV transmission; however, not all are equally competent and mosquitoes belonging to the *Culex pipiens* complex and their hybrids play a central role in virus circulation [7]. While the great majority of mammals are not susceptible to WNV, infected horses and humans sometimes get sick and develop a neuroinvasive disease (<1% of infection cases, [8]). However, humans and horses are considered dead-end hosts because the virus does not replicate enough in these organisms to infect a new mosquito feeding on their blood to continue the cycle [9]. The circulation of WNV among birds may occur silently for several months, or even years, before the spillover event to humans and/or horses occurs [10,11].

West Nile virus is a reemerging zoonosis in Europe, with an increasing incidence in the last decades [11]. Since 2004, there has been evidence of WNV circulation in Spain based on the detection of the virus in mosquitoes [12,13], seropositive resident wild birds [14,15], diseased birds in recovery centres [16,17], and the presence of antibodies in horses [18–20]. West Nile virus associated disease was detected in golden eagles in 2007 [21] and outbreaks in horses have been regularly reported every year since 2010 [22,23]. In the early 2000s, a study of human sera from the province of Seville determined a seroprevalence of 0.6%, being seropositives more common among people from rural areas [24]. The first case of WNV associated disease in humans in Spain was reported in 2004 [25]. Since then, and despite the endemic circulation of WNV in mosquitos, wildlife, and horses, transmission to humans remained low, with two additional cases in 2010 [26] and three more in 2016 [23]. During the 2018 season (the worst WNV season in Europe ever), 2083 cases of human infections (and 181 deaths) were reported among several European and nearby countries [27], however, no human cases and a very small number of horse infections were reported in Spain. Later, in 2020, a large outbreak occurred in Spain with 77 cases of disease in humans and eight deaths. The outbreak predominantly affected the localities of Coria del Río and Puebla del Río (Seville), two villages close to Guadalquivir river surrounded by rice fields. There were also isolated human cases in other villages in the provinces of Seville, Cádiz and Badajoz [23]. Additionally, WNV circulation was also reported in horses in Andalusia, Extremadura, Catalonia, and Valencia [23]. To understand WNV ecology and transmission in Europe, it is critical to identify the factors that may have favoured virus amplification in its enzootic cycle and its spillover into humans, including the identification of the main mosquito and avian species involved in virus amplification. However, such research is difficult because information on WNV circulation in both mosquito and avian populations before and during the epidemic cycle is usually lacking.

This study aimed to: (1) determine which mosquito species played a key role in the amplification of WNV during the outbreak by analyzing the abundance and viraemia in mosquitoes at different localities affected by the outbreak; (2) compare virus prevalence in mosquitoes and antibody prevalence in house sparrows between localities that had WNV outbreaks in horses and/or humans and those that had not; and (3) identify potential avian species that have been involved in virus amplification by analyzing the presence of WNV antibodies in birds of different species. These birds were captured inside the two villages more affected with cases of infection in humans and a third nearby locality without reported cases of infection, neither in humans nor in horses. These allowed us to test whether WNV transmission occurred inside the urban areas or if it was restricted to the wilderness, and humans were infected while visiting natural areas. We also analysed the presence of Usutu virus (USUV) in mosquitoes and USUV antibodies in birds sera because USUV is a closely related flavivirus reported several times in mosquitoes from southern Spain [13,28].

## Methods

### Mosquito survey

**Figure 1.**
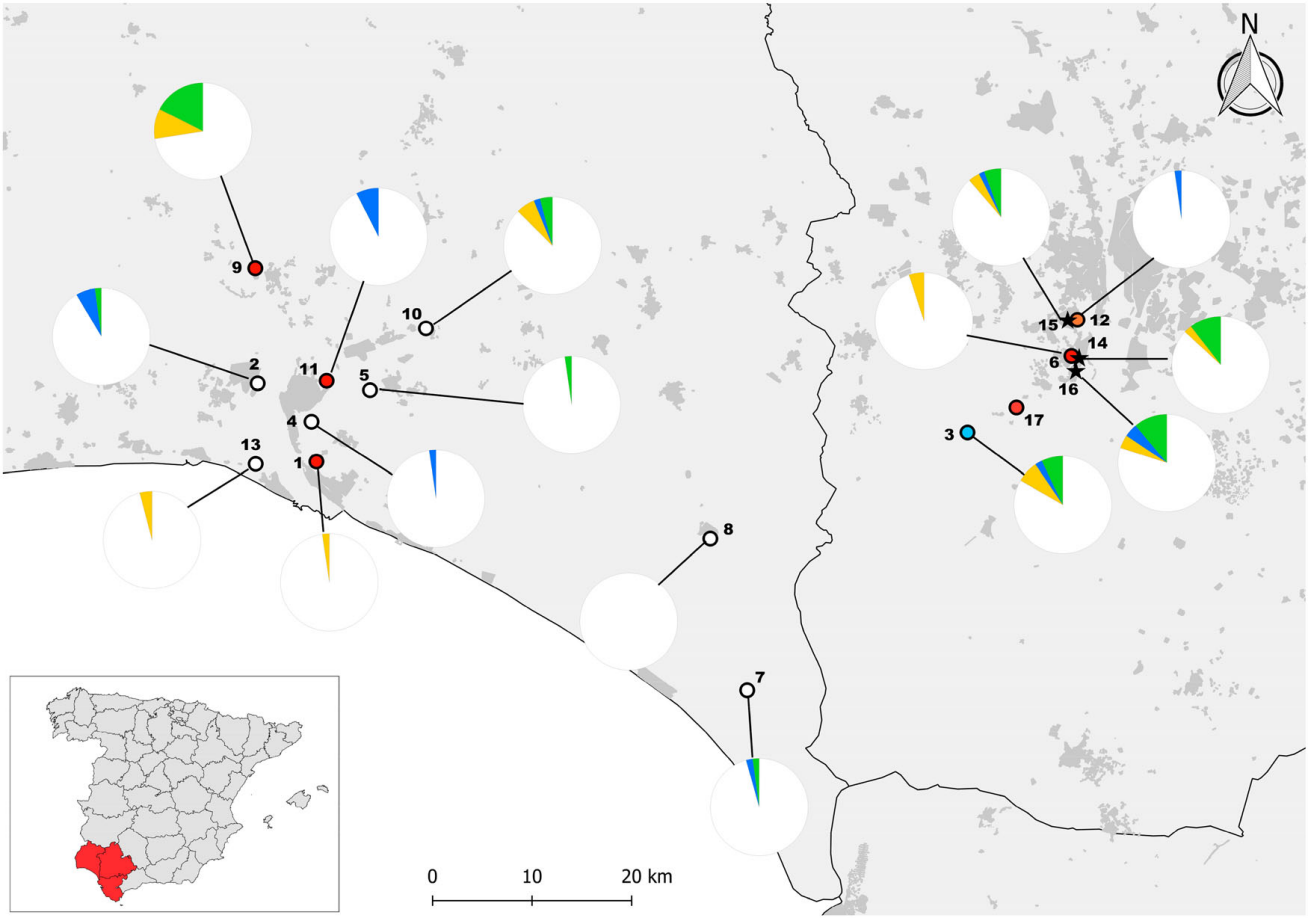
Localities of mosquito collection marked with empty dots for localities without WNV positive mosquito pools and red dots for the localities where at least one pool has been positive for WNV. A blue dot identifies the locality where an Usutu and several WNV positive pools of *Cx. perexiguus* were captured and an orange dot the locality where a WNV infected *Cx. pipiens* and three CxFv infected mosquito pools were captured. Pies indicate the prevalence of WNV (green), USUV (blue) and undetermined flavivirus (orange) antibodies in house sparrows as determined by seroneutralization in 13 localities sampled between July and August 2020, before the detection of WNV human cases, and at three urban localities between September and October 2020 after the outbreak of WNV human cases (localities marked with stars, that correspond from north to south to Palomares del Río, Coria del Río and Puebla del Río). The numbers correspond to the locality names listed in Table 2. Locality 17 corresponds to Cañada de los Pájaros, where only mosquitoes and not house sparrows were captured.

Mosquitoes were trapped at monthly intervals between June and December 2020 at 15 localities spread through the provinces of Seville and Huelva (Figure 1). We placed three BG traps in each locality. They were active for 24 h and baited with approximately 1 kg of dry ice each to generate a continuous flow of CO_2_ at the entrance of the trap. At the four localities closest to Coria del Río and Puebla del Río trapping became weekly from 25 September until 2 December 2020. In addition, from August 25th, we captured mosquitoes with two BG traps and carried out mosquito aspirations in the streets of these two villages to identify the mosquitoes present in the human-inhabited areas. A total of 30 aspiration sessions of approximately 2 h each were done. Adulticide and larvicide treatments in response to the outbreak started by 16 August [23] and were done by different private companies. They involved larvicide treatments with *Bacillus thuringiensis israelensis* in urban scaups and areas with larval concentration 2 km around the urban areas and adulticide treatments with pyrethroids using mainly Ultra Low Volume spraying and Residual Spraying. These samplings inside the urban areas were affected by the adulticide treatments done in the area in response to the outbreak, and consequently, captures were very scarce (Table 1). Mosquitoes were transported in dry ice to the laboratory and stored at −80°C until morphological identification to species level was carried out following Gunay et al. [29] over a chilly table. Specimens belonging to the *univittatus* complex were identified as *Culex perexiguus* based on male genitalia as per Harbach [30]. Mosquitoes were pooled by species, sex, collecting site, and date, in pools of up to 50 individuals. Females with a recent blood meal were stored individually for future blood meal origin analysis and were not included in this study. When several thousands of mosquitoes were captured per trap per night, we identified 500 individuals and determined their weight to the nearest 0.001 g. The total number of mosquitoes was estimated from sample weight, and the proportion of identified individuals of each species was extrapolated for the rest of the sample.

**Table 1.** Number of mosquitoes captured in BG traps and aspirations in the streets of Coria del Río and Puebla del Río between 25th August and the 2nd December of 2020, after the start of the vector control programmes.

	Coria del Río	Puebla del Río
*Anopheles atroparvus*	0	84
*Culex perexiguus*	0	93
*Culex pipiens*	2	39
*Culex theileri*	0	4
*Aedes caspius*	1	6
**Total**	3	226

### Virological analyses

Virological analyses focused on the three species of mosquitoes considered the main vectors of WNV in the study area: *Culex modestus*, *Cx. perexiguus* and *Cx. pipiens* [7,10,12,13]. RNA from mosquito pools was extracted with QIAamp Viral RNA Mini Kit (Qiagen, Valencia, California, USA) following manufacturer instructions. The presence of WNV was tested by two different approaches: (a) a WNV screening using a Real-Time RT–PCR amplifying all the known lineages of WNV [31] and (b) a generic flavivirus screening using a nested RT–PCR following Sánchez-Seco et al. [32] protocol with a modification in the RT–PCR, where QIAGEN OneStep RT–PCR Kit (Qiagen,Valencia, California, USA) was used. Virus infection rates were calculated from mosquito pools using the poolTestR package that estimates infection rates from pooled samples using Maximum Likelihood methods [33].

### Birds capture

**Figure 2.**
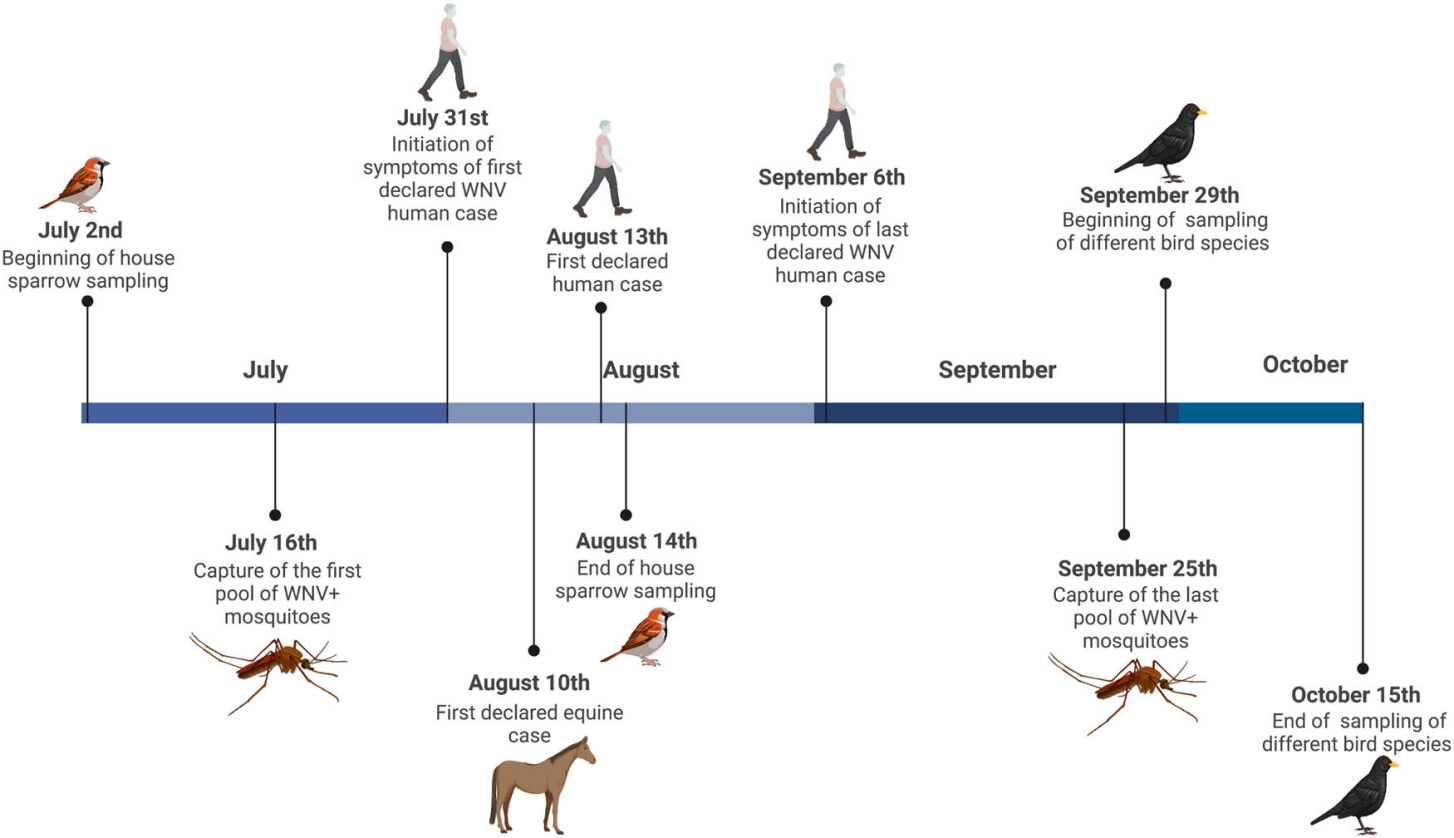
Timeline including the dates when human and horse cases were detected [23,54], the dates when first and last positive cases of WNV mosquitoes were found, and the two periods of bird sampling. Figure Created with BioRender.com.

In 2020, 571 house sparrows were captured between 2nd July and 14th August (before outbreak declaration; Figure 2) at 13 of the 15 localities sampled for mosquitoes, with 40–49 individuals sampled per locality (Table 2). In addition, between the 29th of September and the 15th of October (after the outbreak; Figure 2), 135 house sparrows and 38 birds of other eight different species were captured in parks inside three villages. Two of them, Puebla del Río and Coria del Río, were the two with the highest number of WNV cases in humans. The third, Palomares del Río, was a nearby village without human or horse cases diagnosed.

**Table 2.** Seroprevalence of West Nile virus (WNV), Usutu virus (USUV) or undetermined flavivirus in house sparrows.

	WNV	USUV	Flavivirus	Prevalence
*July–August (before human outbreak)*				
1. Celestino Mutis, Huelva			2.3 (1)	2.3 (43)
2. Corrales, Aljaraque, Huelva	2.1 (1)	6.4 (3)		8.5 (47)
*3. Dehesa de Abajo, Puebla del Río, Sevilla*	7.1 (3)	2.4 (1)	7.1 (3)	16.7 (42)
4. EDAR – Huelva		2.2 (1)	2.2 (1)	4.4 (45)
5. EDAR – Moguer, Moguer, Huelva	2.3 (1)			2.3 (43)
*6. La Hampa, Coria del Río, Sevilla*			5 (2)	5 (40)
*7. El Palacio, Almonte, Huelva*	2.2 (1)	2.2 (1)		4.3 (46)
*8. El Rocio, Almonte, Huelva*				0 (44)
*9. Gibraleón, Huelva*	17.5 (7)		10 (4)	27.5 (40)
10. Granja Escuela, Trigueros, Huelva	4.2 (2)	2.1 (1)	6.3 (3)	12.5 (48)
*11. Los Alamos, Huelva*		7.5 (3)		7.5 (40)
12. La Lagunilla, Palomares del Río, Sevilla				0 (44)
13. Salinas de Punta Umbria			4.1 (2)	4.1 (49)
*September–October (after human outbreak)*				
14. Coria del Río, Sevilla	10.5 (4)		2.6 (1)	13.2 (38)
15. Palomares del Río, Sevilla	5.7 (3)	1.9 (1)	3.8 (2)	11.3 (53)
16. Puebla del Río, Sevilla	11.4 (5)	4.5 (2)	4.5 (2)	20.5 (44)

The overall prevalence of antibodies against flavivirus is indicated together with number of positive individuals and sample size in parenthesis.

Birds were captured using mist nets, individually ringed and their age and sex were determined when possible based on plumage characteristics [34]. From each bird captured, a sample of blood was taken from the jugular vein using sterile syringes. Blood was refrigerated in the field and in a fridge (4°C) overnight until centrifugation the next morning at 4.000 r.p.m for 15 min. Serum was separated from the blood cell pellet and stored at −80°C until analyses.

### Serological analyses

The presence of WNV antibodies was first analysed using the epitope blocking ELISA kit Ingezim West Nile Compac [35], following manufacturer instructions (Ingenasa Spain). Doubtful and positive sera in the ELISA were further analysed by micro-virus neutralization test (micro-VNT) using 96 well plates following Llorente et al. [36]. This test not only confirmed the positivity but allowed us to differentiate serological reactions from cross-reacting flaviviruses. For this purpose we tested sera for virus-neutralization in parallel against three different flaviviruses previously detected circulating in birds in the area: WNV, USUV and Bagaza virus (BAGV) [37]. The employed viral strains in the assays were: WNV E101 (accession no. AF260968), USUV SAAR–1776 (accession no. AY453412), and BAGV Spain/RLP–Hcc1/2010 (accession no. KR108244). Samples showing absence of cytopathic effect (CPE) at 1:10 titre or higher were scored as positives. When titres for one of the viruses were at least 4-fold higher than those observed for the other viruses, the antibodies were considered specific to that virus. Otherwise, the sample was scored as seropositive for undetermined flavivirus because the reaction could be due to antibodies specific for WNV, USUV, BAGV or another putative flavivirus not identified yet in the area. That was the situation for 48 samples, with titres being equal for WNV and USUV for 21 samples, lower for USUV in 11 samples and lower for WNV in 16 samples. None of the bird samples had specific antibodies against BAGV, and since the ELISA test is unspecific for BAGV and some positives may be missed, results for BAGV are not further discussed.

## Results

### Mosquito abundance

**Figure 3.**
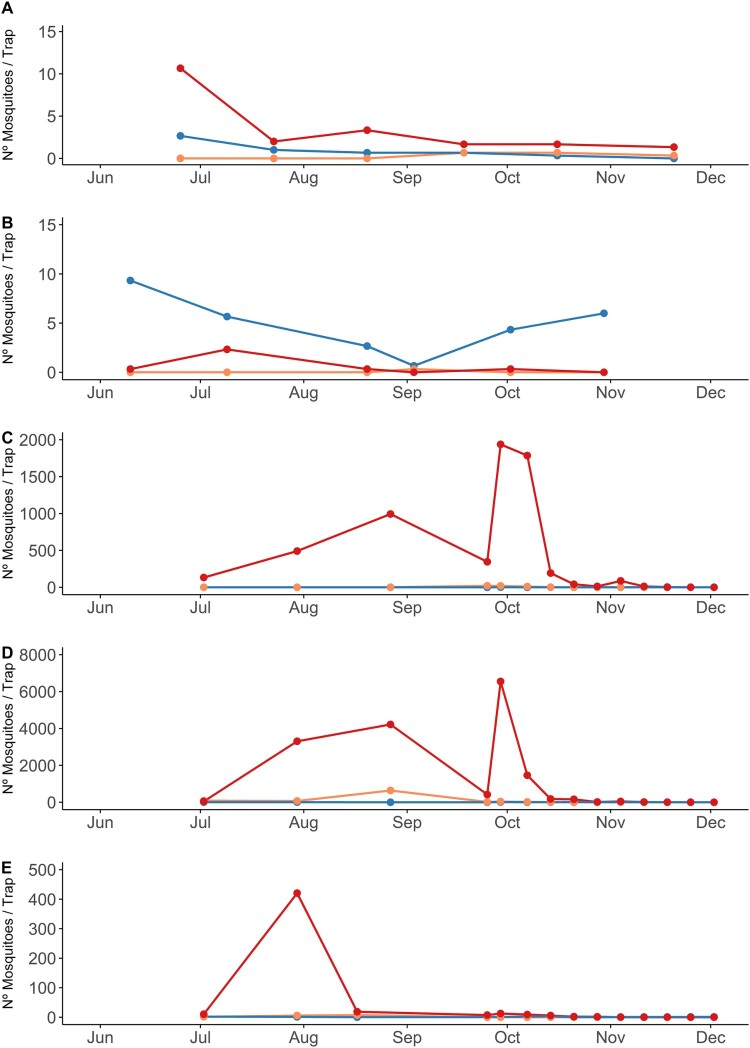
Number of female *Cx. modestus* (orange), *Cx. perexiguus* (red) and *Cx. pipiens* (blue) females captured in BG traps baited with CO_2_ in 2020: (A) Palacio de Doñana (B) Los Álamos (C) Dehesa de Abajo (D) Cañada de los Pájaros (E) La Hampa.

Captures of mosquitoes at the areas close to the main villages affected by the outbreak detected a large proliferation of *Cx. perexiguus* and much lower numbers of *Cx. pipiens* and *Cx. modestus* (Figure 3). In localities in municipalities with human cases, numbers of captures of *Cx. perexiguus* (Figure 3(C–E)) were orders of magnitude over values for localities in municipalities without human cases (Figure 3(A,B)). The mean number (±s.d.) of *Cx. perexiguus* females captured per trap night between July and August 2020 was 538.7 ± 621.7 at Dehesa de Abajo, 2,549.9 ± 3,535.9 at Cañada de los Pajaros and 149.9 ± 221.4 at La Hampa. The numbers were, however, very small for *Cx. pipiens* (Dehesa de Abajo: 0, Cañada de los Pajaros: 3.6 ± 7.5, and La Hampa: 1.2 ± 1.4). The numbers were also very low for *Cx. modestus* (Dehesa de Abajo: 0.1 ± 0.3, La Hampa: 4.9 ± 6.0) with the only exception of Cañada de los Pajaros (265.6 ± 422.4). The captures made inside the villages between the end of August and the beginning of December were very scarce, particularly in Coria del Río where only 3 mosquitoes were trapped (Table 1). In Puebla del Río, *Cx. perexiguus* was the predominant species, followed by *Anopheles atroparvus* and *Cx. pipiens* (Table 1).

### WNV infection in mosquitoes

**Figure 4.**
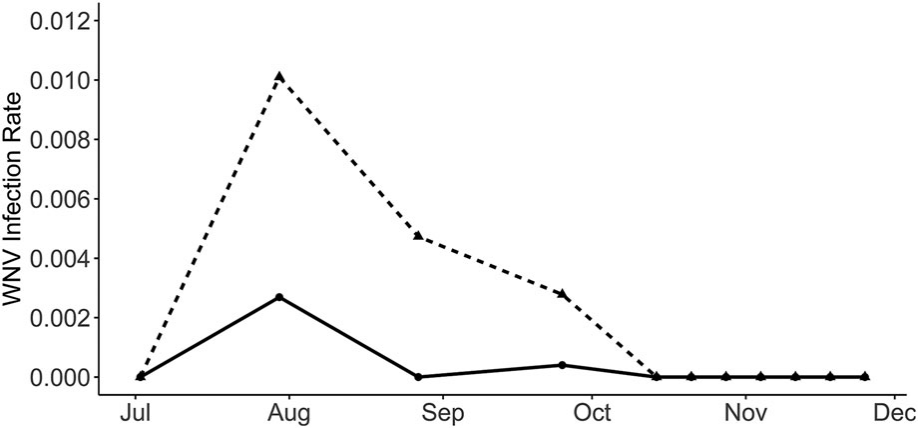
WNV Infection Rate in *Culex perexiguus* captured at Dehesa de Abajo (triangles and dotted line) and Cañada de los Pájaros (points and continuous line) between July and December 2020.

We tested 419 pools of *Cx. perexiguus* (12,513 females), 152 of *Cx. pipiens* (1,563 females) and 75 pools of *Cx. modestus* (881 females) for WNV using the real time RT–PCR and a pan-flavivirus generic RT–PCR. While WNV was identified by Real-Time RT–PCR in 33 *Cx. perexiguus* pools (7.88%) and 1 *Cx. pipiens* pools (0.66%), the generic flavivirus RT–PCR detected WNV only in 19 *Cx. perexiguus* (4.53%) and the 1 *Cx. pipiens* pools (0.66%). In addition, USUV was identified by the generic flavivirus RT–PCR in one pool of *Cx. perexiguus*, and 3 pools (1 of *Cx. pipiens* and 2 of *Cx. perexiguus)* were positive for an insect flavivirus (CxFv) using the same technique. The number of WNV positive pools was much higher when tested by real time RT–PCR than with generic flavivirus RT–PCR (34 vs 20). Also, in the pools that were negative in the generic RT–PCR but yielded positive results in the real time protocol, the Ct value was consistently higher (mean ± s.e. (95% CI): 33.96 ± 1.15 (31.61–36.31)) than for pools positive in the generic PCR (25.25 ± 1 (23.21–27.29), t_33 _= 5.70, *p* < 0.0001). This is likely due to a higher sensitivity of the real-time over the generic RT–PCR. The real time RT–PCR allowed to detect the first positive pool 15 days earlier than the generic RT–PCR (16 July 2020 vs 30 July 2020), and 29 days earlier than the first laboratory confirmation of a WNV human case [23] (Figure 2). WNV positive mosquitoes were detected at seven different localities (Figure 1), 3 in municipalities with human cases (Coria del Río and Puebla del Río), 3 at municipalities with only horse cases (Gibraleón and Huelva) and one at a municipality without cases of WNV reported in humans or in horses (Palomares del Río). Intensity of infection in mosquitoes peaked at the end of July, and the last *Cx. perexiguus* positive pool was detected by 25 September (Figure 4).

### Prevalence of antibodies in house sparrows before the outbreak

There were WNV seropositive house sparrows at six localities (Table 2). Prevalences ranged between 2.1% and 17.5% and measured WNV-neutralizing antibody titres ranged from 1:10 to 1:1240. The highest seroprevalence was found at Gibraleón, in a mixed area with houses, orchards and avian (chicken and turkey) farms. At Gibraleón, WNV was also detected in mosquitoes and horses, but no human cases were reported. Dehesa de Abajo, a freshwater pond near the main human outbreak area where the largest amount of *Cx. perexiguus* mosquitoes were captured, also presented high seropravelence. The other four localities with WNV seropositive sparrows (Aljaraque, Almonte, Moguer, Trigueros) did not report human cases, although one (Trigueros), reported equine WNV cases. In addition, in Aljaraque, Almonte, Coria del Río, Huelva and Trigueros there were USUV seropositive house sparrows.

### Prevalence of antibodies in urban birds after the outbreak

There were WNV seropositive house sparrows in the three urban localities sampled after the human outbreak. The highest seroprevalences were found in birds from Puebla del Río (11.4%) and Coria del Río (10.5%), but also 5.7% of birds from Palomares del Río had WNV antibodies (Table 2). Moreover, we detected USUV seropositive house sparrows in Palomares del Río and Puebla del Río. The highest seroprevalences for WNV, considering only avian species with at least five individuals sampled, were found in *Turdus merula* (40.9%), *Streptopelia decaocto* (20%) and *Passer domesticus* (8.9%, Table 3). Considering overall flavivirus seroprevalence, values reached 90.9%, 40% and 14.8% in these three species, respectively (Table 3).

**Table 3.** Seroprevalence of West Nile virus (WNV), Usutu virus (USUV) or undetermined flavivirus (Flavivirus) antibodies in different avian species captured in the urban areas of three different villages after the 2020 West Nile virus human outbreak in Andalusia.

Avian species	WNV	USUV	Flavivirus	Prevalence
*Bubulcus ibis*	100 (1)			100 (1)
*Columba livia*	100 (1)			100 (1)
*Estrilda astrild*				0 (1)
*Ficedula hypoleuca*				0 (3)
*Passer domesticus*	8.9 (12)	2.2 (3)	3.7 (5)	14.8 (135)
*Streptopelia decaocto*	20 (1)		20 (1)	40 (5)
*Sturnus unicolor*		25 (1)		25 (4)
*Turdus merula*	40.9 (9)	18.2 (4)	31.8 (7)	90.9 (22)
*Upupa epops*	* *		100 (1)	100 (1)

The overall prevalence of antibodies against flavivirus is indicated together with number of positive individuals and sample size in parenthesis.

## Discussion

The results of this study indicate that WNV intensively circulated during the spring-summer of 2020 in the urban areas of Coria del Río, Puebla del Río and Palomares del Río. These results suggest that some human infections would be produced by mosquito bites within the villages and not necessarily while visiting natural areas or nearby rice fields. The area of circulation of the virus was also larger than expected. It included both localities where WNV disease was detected only in horses (i.e. Gibraleón) and localities without reports of WNV disease in either horses or humans (i.e. Palomares del Río) [38]. According to the seroprevalence data in house sparrows, WNV circulation was more intense in Puebla del Río, and decreased from Coria del Río to Palomares del Río. Such differences in the prevalence of antibodies in house sparrows were associated with the relative incidence of human cases among the population from the three villages [23]. Figuerola et al. [14] found that larger bird species have a higher prevalence of WNV antibodies. According to this finding, the prevalence of WNV antibodies in larger urban bird species such as European blackbirds (*Turdus merula*) was much higher than in the smaller house sparrows. The European blackbird is an ecologically similar species phylogenetically related to the American robin in north America. Both species have similar sizes (95.85 g vs 77.3 g, [39]) and have important populations in cities [40]. In addition, both are bitten by *Cx. pipiens* more than expected from their relative frequency in the avian community [6,41], and have a high host competence as judged by the high viremias they reach, facilitating mosquito infection. Information on the avian species involved in the amplification of WNV in urban areas of Europe is scarce and mainly derived from reports of dead birds, but not from WNV serology after epidemics. Such studies have highlighted the role of corvids, and in particular magpies in WNV transmission and its high susceptibility to the virus [42,43]. However, magpies are not very frequent in the area of this outbreak [44]. Furthermore, the most common corvids in the area are jackdaws (*Corvus monedula*) but this species presents very low WNV antibody prevalences in comparison to other avian species in this area (see Figuerola et al. [14]).

An important question is why so many cases of WNV in humans occurred in 2020. Firstly, changes in vector/host communities involved in transmission may help to explain this unusually virulent outbreak. Previous studies in the province of Seville found an average seroprevalence in humans of 0.6%, with a higher seroprevalence in rural areas [24]. In this area, *Cx. perexiguus* may play a key role in the transmission of WNV [10,13], and WNV seroprevalence in house sparrows in 45 different localities was positively related to *Cx. perexiguus* abundance [45]. Further support for the importance of this mosquito species in the transmission of WNV to birds derived from epidemiological models [46] that indicate that WNV Basic Reproductive Number is much larger when *Cx. perexiguus* is present in the community. According to our results, the important proliferation of *Cx. perexiguus* in nearby areas might be the trigger of the outbreak. Rice fields constitute the main breeding habitat of *Cx. perexiguus* in the area [47], and the lack of control measures against mosquitoes in the last years have probably favoured the proliferation of this species. The entomological inspections done in the area immediately after the WNV outbreak declaration found high numbers of *Cx. pipiens* larvae in the sewers of Coria del Río and Puebla del Río [48]. In contrast, no *Cx. perexiguus* larvae were found in such urban infrastructures ([48] and own unpublished data). Based on this data, we propose that *Cx. perexiguus* played a central role in the enzootic transmission of WNV in the areas surrounding the villages, and also inside some of the villages, while *Cx. pipiens* may have acted as a bridge vector favouring the transmission of the virus to humans. The blood meal analyses done until now in the area suggest that humans are not preferred hosts for *Cx. perexiguus* [10], although such studies were done in natural areas where humans were not present and consequently their feeding preferences in urban areas may change and needs to be evaluated. High summer temperatures and winter and spring rainfall [49] may have also favoured the proliferation of *Cx. pipiens* and *Cx. perexiguus* inside the villages and the amplification of WNV in 2020 [27,50]. In addition, the restriction of movements due to the COVID19 emergency may have favoured the breeding of mosquitoes in buckets, barrels, and other structures susceptible to accumulating water in urban and suburban areas. Although the strain circulating in 2020 was closely related to other strains already detected in Europe [51], we cannot rule out that the higher number of human clinical cases was the result of higher intrinsic pathogenicity of 2020 WNV strain. This possibility is being examined by specific *in vivo* experimental infection studies that are currently ongoing (M.A. Jiménez-Clavero, unpublished data).

In addition, we also detected active circulation of USUV, with positive mosquitoes and a high seroprevalence in different localities in the study area. The detection of USUV calls for reinforcing the surveillance to detect potential clinical infections in humans and prevent future outbreaks. Integrated surveillance and control of mosquitoes is necessary to prevent new WNV (and other flavivirus) outbreaks in the studied area. It is advisable to incorporate environmentally friendly control programmes to reduce mosquito breeding inside the villages and in the neighbourhood of the villages, including the rice-growing areas close to inhabited areas. Vector and virus surveillance may provide early warning signals of unusual mosquito abundance and virus proliferation and inform control programmes, allowing adaptative management of vector populations. Similar programmes are implemented in different areas of North America and Europe i.e. [52,53] and have allowed the development of early warning protocols to inform vector control programmes and reduce WNV impact. Considering the large history of WNV circulation in Andalusia and the potential for future outbreaks it is highly advisable to implement such a programme in the localities affected by the 2020 WNV outbreak.

Summarizing, this study uses a One Health approach to monitor the circulation of WNV in the area of the largest WNV outbreak that occurred in Spain ever. Overall these results highlight the importance of implementing adequate mosquito surveillance protocols in Western Andalusia to prevent new WNV outbreaks.

## Data Availability

The data that support the findings of this study are available from the corresponding author, JF, upon reasonable request.
